# The Rutgers Integrated Care Evaluation (RICE) Research Framework: An Innovative and Rigorous Set of Methods to Evaluate Integrated Care Programs

**DOI:** 10.5334/ijic.7715

**Published:** 2024-09-23

**Authors:** Jamey J. Lister, Holly H. Lister, Kristen G. Powell, Shannon P. Cheung, N. Andrew Peterson, Anna Marie Toto, Stephanie C. Marcello

**Affiliations:** 1 School of Social Work, Rutgers, The State University of New Jersey, USA; 2 Center for Integrated Care, Behavioral Research and Training Institute, Rutgers University Behavioral Health Care, USA; 3 Northeast & Caribbean Prevention Technology Transfer Center, Center for Prevention Science, School of Social Work, Rutgers, The State University of New Jersey, USA

**Keywords:** methodology, integrated care, medical, behavioural health, research, evaluation

## Abstract

**Introduction:**

Integrated care programs that prioritize comprehensive service delivery for behavioural health and medical conditions have the potential to improve patient outcomes. Few programs, however, use data-driven methods to guide program evaluation and implementation, limiting their effectiveness, as well as the scope of findings in the research literature.

**Purpose:**

To address these gaps, we describe an innovative and rigorous evaluative research framework: the Rutgers Integrated Care Evaluation (RICE) Research Framework, designed to be tailorable across conditions and care settings.

**Method:**

The RICE Research Framework is guided by two core concepts: (1) an approach built on engaging as equal partners and (2) data source triangulation. For the former, the approach relies on multiple teams (Project, Clinical Site, Evaluation, and Consumer) working in collaboration. While teams have specific roles, all teams engage frequently as equal partners to facilitate performance and advance research deliverables. For the latter, we provide a template with recommended primary and secondary data sources with areas of focus, applicable methods, and samples. These sources, when used in combination, can guide implementation, advance replicability, develop/refine health care programs, and foster dissemination of scientific findings.

**Conclusions:**

We recommend clinicians and scientists implement the RICE Research Framework to enhance their integrated care programs.

## Introduction

Integrated care (IC) programs that provide comprehensive services for both behavioural health (e.g., substance use, anxiety, depression) and medical (e.g., chronic heart failure, diabetes) conditions in a single location with a shared team of providers for both areas of specialization have been shown to improve patient outcomes such as reduced hospital admissions and re-admissions, and improved quality of life [1,2,3,4]. Improved outcomes are reflected across several populations, including children and adolescents [5], adults [1,2,3] and medically underserved groups [4,6].

Along with the empirically demonstrated benefits of IC, there has been a rise in recent decades in large funding sponsors in the United States (e.g., Health Resources and Services Administration/HRSA, Substance Abuse and Mental Health Services Administration/SAMHSA) supporting IC initiatives. However, programs funded to expand the health workforce or implement health services typically prioritize meeting baseline performance measure deliverables as required by the funding sponsor, but do not emphasize or produce standalone research or independently determined aims grounded in scientific inquiry. As a result, there is likely to be considerable variability in the degree to which rigorous, data-driven methods are used to guide the evaluation and implementation of IC programs at multiple levels (i.e., patient, provider, clinic, and community). While some IC programs disseminate findings on processes and outcomes in scientific journals [7,8], the imperative to advance knowledge beyond technical reports to funding sponsors may be mixed. This may especially be the case when the projects are led by practitioner-scientists whose career performance and job security is assessed largely on their ability to bring in large-scale, multi-year grants that expand programming. Overall, this may minimize the number of peer-reviewed publications produced by such programs, hindering knowledge development, clinical training and programmatic development, and replicability. Complicating matters, IC-specific evaluative research frameworks are scarce. Many existing frameworks prioritize economic evaluation to justify cost-effectiveness [9], rather than focusing on patient outcomes, provider competency, care coordination changes, or reductions in morbidity among medically underserved urban or rural communities served by their program(s). In addition, existing research frameworks emphasize the evaluation of IC programs specific to certain populations (e.g., hospitalized older adults, patients in palliative care, paediatric populations) [5,10], which excludes a rising number of programs integrating care for other populations such as those with substance use disorders or other mental health disorders [11].

While program evaluation is traditionally required by health and human service programs to improve the quality of integrated care programs, it also should rigorously highlight how the program is meeting patient, provider, and/or community needs. Herein, we argue that IC evaluation research should tackle more than what is required by the funding sponsor to identify unmet needs from all actors involved in the health care system. Furthermore, we outline more rigorous scientific approaches be utilized to create findings that can generalize across settings and inform broader changes to the health care system. Thus, there is a need for a comprehensive, tailorable, and generalizable IC evaluation research framework that relies on data-driven approaches.

## Purpose

The lack of evaluative standards in IC research has left many programs to generate idiosyncratic methodologies. These approaches lack clear standards for measuring and analysing effectiveness beyond their respective funding sources’ required performance measures, which can vary from initiatives even housed within the same centre or bureau of the funding sponsor’s programming. A set of tailorable standards to guide IC program evaluation is critical to facilitate the development of organized systems of care [12,13] and to improve patient outcomes [1,2,3,14]. To address these gaps, we describe an innovative and rigorous evaluative research framework: the Rutgers Integrated Care Evaluation (RICE) Research Framework. The RICE Research Framework is currently being used as an evaluative research tool in multiple HRSA-funded IC programs that embed behavioural health and substance use services into primary medical care in medically underserved areas [15], as well as guiding evaluations for SAMHSA-funded care coordination programs integrating mental and physical health services for patients receiving treatment for substance use. Importantly, this research framework is designed to be tailorable across care settings and health conditions, making it applicable in a range of settings (e.g., academic medical centres, community health centres, clinical training programs, federally qualified health care providers).

## Method: The RICE Research Framework

The RICE Research Framework is guided by two core concepts. These concepts represent a novel evaluation research framework that was designed iteratively over time by the framework’s authors during several collaborative IC program implementations that have demonstrated sustained program quality and outcomes.

**Figure 1:**
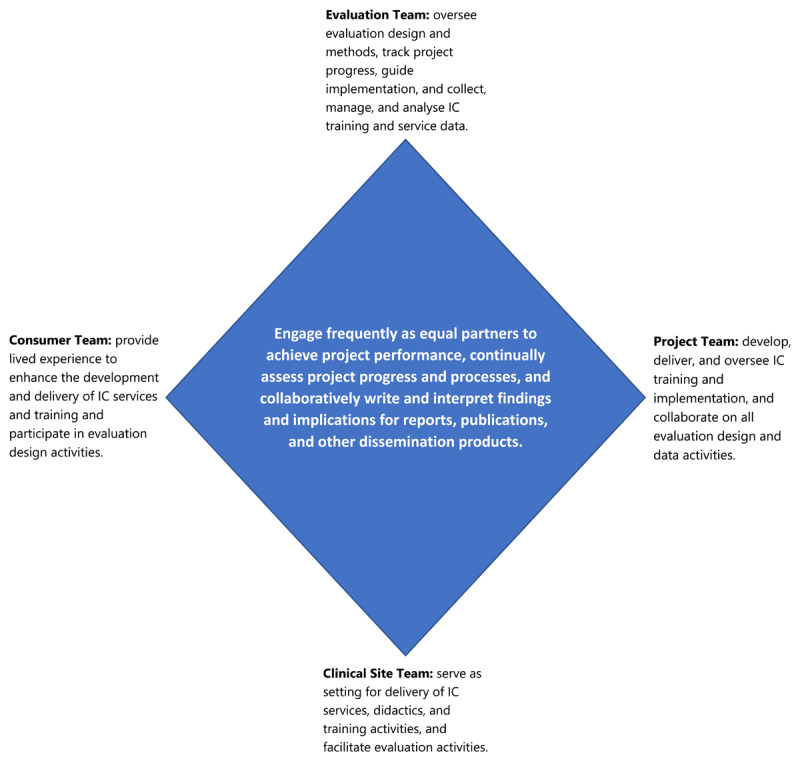
The RICE Research Framework: Engaging as Equal Partners Approach.

The first core concept of the research framework is utilizing an “engaging as equal partners approach” comprised of four teams (see Figure 1). There is a Project Team that develops, delivers, and oversees IC clinical training and implementation activities. More specifically, the Project Team coordinates specific IC didactic curricula for medical/behavioural health trainees to support their placements within primary care sites implementing an IC model. The Project Team also closely collaborates on all evaluation design and data activities and acts as liaison between IC medical/behavioural health trainees and their clinical placements. There is a Clinical Site Team where IC services are implemented, delivered, and medical/behavioural health trainees providing clinical activities are placed. For sites in which medical/behavioural health trainees are placed for IC clinical experiences, the Clinical Site Team works closely with the Project Team to ensure successful training. Data from medical/behavioural health trainees, clinical site leaders, patients (and other consumer groups) is gathered from these clinical sites and the Clinical Site Team facilitates evaluation design activities. There is an Evaluation Team who is responsible for overseeing the evaluation research design and methods, collecting, managing, and analysing all IC training and service data, tracking project performance (process, progress), and guiding changes to project implementation. There is also a Consumer Team who provides input (i.e., through their lived experience as peers, recovery coaches, patients, and/or community members) to enhance the development and delivery of IC services and training and participates in evaluation design activities. This equal partnering approach uses clearly defined roles for each team as described above. The Project Team and Clinical Site Team meet on a regularly scheduled basis and as needed to track medical/behavioural health trainee performance and feedback within the program, and to gather Clinical Site Team feedback. The Evaluation Team and Clinical Site Team meet via scheduled meetings and as-needed communications to coordinate and collect primary data from clinical site leaders and providers, as well as to transfer data for secondary data sources such as electronic medical record (EMR) data that can identify changing patterns in care coordination and utilization at the clinic before and after the program’s implementation. The Evaluation Team and Project Team meet via “open-door” communications on an ongoing basis and in monthly meetings to track medical/behavioural health trainee program process, data collection, and to overview all aspects of the evaluation research progress. The Consumer Team engages with the Project Team during development to emphasize IC needs and considerations. During evaluation design, the Consumer Team can provide input to the Evaluation Team regarding the research design, co-collect primary data, and participate alongside all other Teams to interpret findings.

The purpose of this “engaging as equal partners approach” is to increase the frequency of engagement, improve effectiveness and efficiency in efforts to identify and assess performance metrics, use data to inform all project decisions and assess program needs, and to work jointly to disseminate information learned to clinical sites, community stakeholders, and other institutions wishing to replicate similar dual clinical implementation and clinical training programs. Last, data-driven measures are written up collaboratively for reports, publications, and other dissemination products to enhance replicability and knowledge development in the larger scientific community.

Benefits of this multi-pronged equal partner approach to evaluation research are stronger designs, more robust data collection and analysis, translatable results, and improved trust of recommendations when engaging with consumer populations during the planning process [16,17,18]. Within this approach, each partner is equally valued and can provide expertise in different areas of knowledge dissemination about program development, implementation, and evaluation. To illustrate this, in one of the ongoing HRSA-funded projects on IC for substance use and other behavioural health disorders [15], the Evaluation Team leads design of the methodology, data collection, and analysis, while the Project Team disseminates information about the efficacy and/or potential for replicability of their IC training model. The Clinical Site Team and Project Team are then able to provide a unique interpretation of how the findings inform improvements in healthcare systems similar to the Clinical Site Teams’. In these collaborations, the Evaluation Team is able to bring advanced mixed methods (quantitative and qualitative) and analytic approaches, such as the use of validated self-report surveys assessing attitudes and practice experiences relevant to IC delivery, data cleaning and cross-walking of EMR data, and development and administration of rigorous focus group and in-depth interview guides and thematic analysis of participant data to demonstrate barriers and facilitators of IC implementation. Furthermore, publications involving members of the author team have used similar partnering approaches to enhance credibility of findings by engaging consumers with lived experience during research design and interpretation [19,20].

The research framework’s second core concept is striving for data source triangulation (see Table 1), which proposes IC programs measure processes and outcomes across multiple data source domains relevant to IC program functioning [12] using primary and secondary sources. We recommend inclusion from four sample sources using primary data: leaders at clinical sites, providers and medical/behavioural health trainees at clinical sites, patients and consumers from their social networks (e.g., families and caregivers) being served by clinical sites, and quality improvement data from the Project Team and/or Clinical Site Teams.

**Table 1:** The RICE Research Framework: Data Source Triangulation.


SOURCE	PRIMARY DATA				SECONDARY DATA	
	
	CLINICAL SITES			PROJECT TEAM	CLINICAL SITES	
	
	SITE LEADER DATA	PROVIDER AND TRAINEE DATA	PATIENT AND CONSUMER DATA		PATIENT DATA	COMMUNITY DATA

**Areas of Focus**	Attitudes about IC, key health conditions, and patient populations	Attitudes about IC, key health conditions, and patient populations	Barriers and facilitators (individual, system) of IC program access	Implementation challenges and strategies	Behavioural health disorder rates and care patterns (using procedure codes)	Sociodemographic characteristics from public sources (e.g., US Census Bureau)
	Barriers and facilitators (individual, system) of implementing and sustaining IC programs	Demographic and training backgrounds	Patient, family and caregiver preferences for IC and service delivery	Demographic and training backgrounds	Disparities or population-specific needs by race, gender, insurance, etc.	Medically underserved area definitions
		Clinical competencies and activities				Treatment and outcome data (health service databases)

**Methods**	Focus groups and in-depth interviews	Focus groups and in-depth interviews	Focus groups and in-depth interviews	Real-time continuous quality improvement cycles	Extraction, transfer, and cleaning of medical records	Extraction, cleaning, and linking of public data sources
	Surveys	Surveys	Surveys	Surveys	Pretest/posttest or time series designs to assess changes over time	Pretest/posttest or time series designs to assess changes over time
	Needs assessments and feasibility analyses	Pretest/posttest designs to assess changes over time	Needs assessments			


With regard to the methods and focus of data source triangulation, we recommend different but overlapping strategies. For clinical site leaders, we recommend collecting data regarding attitudes about IC, behavioural health and medical conditions treated, and predominant patient populations served (e.g., insurance type, gender, race/ethnicity, etc.). For providers and medical/behavioural health trainees, we recommend collecting data on clinical competencies and activities. For clinical site leaders, providers, and medical/behavioural health trainees, we recommend collecting data from focus groups and in-depth interviews during and after the end of their training program to anticipate and retrospectively identify barriers and facilitators of IC service delivery in real-world clinical sites. For patients and members of their social networks, we recommend collecting data related to preferences for service delivery, as well as barriers to and facilitators of IC access that address acceptability, availability, accessibility, accommodations, and affordability. For the Project Team, we recommend collecting process data, such as implementation challenges and strategies that can be used to guide quality improvement.

We encourage Evaluation Teams to use multiple and mixed methods approaches to collect primary data involving focus groups, in-depth interviews, needs assessments, feasibility analyses and survey data. We recommend use of existing and validated measures when collecting survey data, in addition to development of customized data collection tools when and if existing measures do not fit the aims of the IC evaluation. When possible, it is advised to collect data at repeated time points to assess whether there are pre/post changes in attitudes, barriers, or clinical activities that occur after IC program implementation at the clinical site(s) and/or after health educational activities regarding IC. We also propose routine collection of two secondary data sources, which include clinical site data using electronic and paper chart medical records (e.g., demographics, behavioural health diagnoses, treatment procedure codes), as well as community data (e.g., community-level treatment provider supply and behavioural health service utilization) from public health databases that can be linked with public directories and resources to characterize the sociodemographic and treatment circumstances among the communities where clinical sites are located. Similar to primary data methods, we recommend that the Evaluation Team create longitudinal data sets from these sources that ideally involve multiple years of data retrospective to the implementation of the IC program, at least one year of data during implementation, and multiple years of data post implementation, to ensure an ability to investigate (i.e., through statistical approaches such as interrupted time series analyses) pre/post changes related to IC implementation in both the clinical site EMR data and the community-level data. To address implementation barriers, a continuous quality improvement (CQI) cycle is used with the following concepts across data source domains: feedback from monthly meetings between the Project and Evaluation Teams; analysis of Project Team meeting minutes by the Evaluation Team; open-door email policy between Project, Evaluation, and Clinical Site Teams to facilitate and expedite project communication and collaboration; data collected from clinical site leaders (e.g., infrastructural barriers within the system) and providers (e.g., beliefs about substance use that may act as barriers to care) through focus groups and surveys that are analysed and submitted to the Project Team through interim reports.

## Conclusions

The RICE Research Framework represents an innovative and rigorous set of methods that can improve the quality of evaluation research for IC programs that serve a range of patient populations and provide medical and behavioural health education. This research framework is a departure from existing IC evaluation frameworks, in that, rather than focusing on the question of whether an IC program is cost-effective, the RICE Research Framework emphasizes IC program processes, provider attitudes and competency, feasibility and acceptability among consumers, community needs and clinical outcomes. This includes collecting data on trainee competencies and clinical activities, barriers and facilitators of IC delivery in specific communities and sites, and patient-level data related to accessibility and acceptability. In addition, RICE provides a research framework for evaluating IC programming for special populations who haven’t been the historical focus of IC (e.g., people reporting heavy or disordered use of substances), thus allowing assessment of organizational and provider-level beliefs and attitudes about policies and patient outcomes specific to the needs of that target population. Many IC programs have used some form of evaluation to assess patient and provider outcomes, but few standardized approaches to IC program evaluation have been put forth. Like existing frameworks and past IC evaluation studies [4,21], we prioritize the use of data-driven decision making through the triangulation of primary and secondary data, collected from multiple sources. We recommend IC teams consider implementing a localized version of the research framework, tailored to their setting and target populations, to focus their primary data collection from key samples at clinical sites (site leaders, providers, medical/behavioural health trainees, patients and their families/caregivers, as well as the Project Team), while also curating secondary sources (EMR data, community health data). Importantly, the framework is designed to offer a flexible roadmap for evaluating IC programs that does not aim for the goal of uniform program replication. Conversely, it emphasizes the ability to tailor evaluations to meet the needs of different patient populations, treatment settings, types of IC approaches, and treatment regions. While the RICE Research Framework has origins with IC training grants, it has also been used for IC service implementation grants and is designed to be tailorable across IC approaches and settings.

Future research should conduct an empirical analysis of the RICE Research Framework’s impact on program quality and outcomes across treatment settings and populations as a next step to inform how primary and secondary data should optimally be used to improve implementation. Furthermore, we encourage all IC evaluation researchers to emphasize clear and consistent data management protocols given the volume and scope of data involved when evaluating IC programs – creation of a standardized data management protocol may be an area of benefit for a range of IC programs.

In summary, the RICE Research Framework is a methodological tool that can support collaborative IC teams providing health education, training, and service implementation. The tool has the potential to increase effectiveness and advancement of IC, under the overarching goal of improving patient outcomes. We encourage cross-sector teams to use this flexible framework to better ensure that evaluative research data is generated in manners that inform implementation strategies, enhance feasibility, improve program effectiveness, and foster dissemination of scientifically relevant findings.
